# Rapid flowing cells localization enabled by spatiotemporal manipulation of their holographic patterns

**DOI:** 10.1063/5.0222932

**Published:** 2024-09-10

**Authors:** Zhengzhong Huang, Zhe Wang, Daniele Pirone, Vittorio Bianco, Lisa Miccio, Pasquale Memmolo, Liangcai Cao, Pietro Ferraro

**Affiliations:** 1 Institute of Applied Sciences and Intelligent Systems “E. Caianiello”, Italian National Research Council (ISASI-CNR), Italy; 2 Department of Precision Instrument, Tsinghua University, Beijing 100084, China; 3 Dipartimento di Ingegneria Chimica, dei Materiali e della Produzione Industriale, Università degli Studi di Napoli Federico II, Piazzale Vincenzo Tecchio 80, 80125 Napoli, Italy

## Abstract

Lab-on-a-Chip microfluidic devices present an innovative and cost-effective platform in the current trend of miniaturization and simplification of imaging flow cytometry; they are excellent candidates for high-throughput single-cell analysis. In such microfluidic platforms, cell tracking becomes a fundamental tool for investigating biophysical processes, from intracellular dynamics to the characterization of cell motility and migration. However, high-throughput and long-term cell tracking puts a high demand on the consumption of computing resources. Here, we propose a novel strategy to achieve rapid 3D cell localizations along the microfluidic channel. This method is based on the spatiotemporal manipulation of recorded holographic interference fringes, and it allows fast and precise localization of cells without performing complete holographic reconstruction. Conventional holographic tracking is typically based on the phase contrast obtained by decoupling the calculation of optical axial and transverse coordinates. Computing time and resource consumption may increase because all the frames need to be calculated in the Fourier domain. In our proposed method, the 2D transverse positions are directly located by morphological calculation based on the hologram. The complex-amplitude wavefronts are directly reconstructed by spatiotemporal phase shifting to calculate the axial position by the refocusing criterion. Only spatial calculation is considered in the proposed method. We demonstrate that the computational time of transverse tracking is only one-tenth of the conventional method, while the total computational time of the proposed method decreases up to 54% with respect to the conventional approach. The proposed approach can open the route for analyzing flow cytometry in quantitative phase microscopy assays.

## I. INTRODUCTION

The astonishing development of bio-microfluidics technology strongly demands substantial improvements in multifunctional tools for characterization, monitoring, and manipulation in microfluidic environments. Lab-on-a-Chip (LoC) devices emulate the functionalities of a modern analysis laboratory as a compact system, which is realizable at contained costs.^1^ To provide imaging with a reduced number of optical components, LoC devices often exploit the flow of samples in a microfluidic environment. This has led to the development of smartly engineered devices for accurate microfluidic control, providing imaging with novel features and improved capabilities.^2,3^ The cost-effective solutions in the current trend of miniaturization and simplification of imaging flow cytometry (IFC)^4^ will allow highly innovative future scenarios.

Several applications have been developed for retrieving the three-dimensional (3D) trajectories of single cells inside a microfluidic channel (MFC). For this purpose, various algorithms of particle tracking can be adopted.^5^ Particle 3D tracking is the key technology for quantitative analysis in LoC cell diagnostic systems. Typically, detecting and following individual particles in a time series of images is referred to as single-particle tracking. Particle imaging velocimetry (PIV) and particle tracking velocimetry (PTV) allow fluid velocities to be measured quantitatively across volumetric regions and are important techniques for fluid visualization and understanding.^6–11^ However, observing 3D trajectories of particles is in general a challenging task in classical microscopy owing to the limited imaging depth of field of commercial optical microscopes, which represents a serious drawback for the analysis of time-lapse microscopy image data.^12^ The existing widely used 3D particle velocimetry acquisition setups inevitably suffer from a trade-off between depth of field and exposure for fast motions, which in turn increases the uncertainty for depth localization. Such a limitation has been overcome by digital holography (DH) and quantitative phase imaging (QPI) in flow cytometry.

As a 3D, noncontact, nondestructive microscopy technique, DH is a full-field and label-free imaging technique that is able to provide quantitative phase-contrast maps (QPMs) with high-precision optical measurements.^13–18^ In recent years, many improvements have been made, either in optical arrangements or in numerical image processing algorithms, in order to make DH a high-throughput imaging instrument to furnish diagnostic tools in many fields, including optofluidics,^19,20^ biomedical microscopy,^21,22^ tumor cell detection,^23^ and 3D cell morphometry.^24,25^ The interferometric principle of a DH microscope has facilitated its first miniaturization into LoC devices.^26,27^ Moreover, the DH's powerful capabilities in micron-scale cell imaging matches well with the requirements of IFC systems.^28^ In fact, holographic imaging flow cytometry (HIFC) takes advantage of DH to provide *a posteriori* multiple refocusing capability.^29^ For example, this property has been exploited to realize holographic particle tracking velocimetry.^30–33^ Holographic particle tracking is often used in different branches of bio-microfluidics.^34–38^ Conventional holographic tracking methods are typically composed of two main steps: (i) numerical refocusing, for retrieving the position of the targets along the optical axis, and (ii) evaluation of the transverse position of the refocused object after QPM reconstruction.^12^ Several contributions in recent years have been proposed in the literature for investigating increasingly accurate refocusing strategies^39–43^ as well as suitable two-dimensional (2D) localization approaches for QPMs.^44,45^ With the increasing data throughput in HIFC, a fast and precisely calculated method for particle tracking is required for high-throughput data collection.

In conventional holography, tracking is performed mainly on the complex amplitude from holograms.^12^ Hence, computing resources and time consumption may increase because all the frames need to be processed and reconstructed. Here, we propose a new strategy to speed up 3D holographic particle tracking of single cells flowing inside a HIFC system. In this strategy, a spatiotemporal operation of the recorded digital holographic fringes is performed, in order to extract the 3D position information of each cell without performing numerical reconstruction. Transverse positions are directly located by morphological calculation based on the interference pattern after spatiotemporal filtering, thus avoiding any phase retrieval. The axial positions are instead calculated by means of a spatiotemporal phase shifting. Experimentally, the proposed method achieves a localization error lower than 1.5 *μ*m by 54% of the computational time with respect to the conventional method. Here, we present a novel approach that could open new perspectives for enhancing the throughput by 3D rapid volumetric imaging.

## II. RESULTS AND DISCUSSION

The performances of the proposed spatiotemporal operations tracking method is discussed in this section. As described in Methods, the first step is background elimination, which is applied to the down-sampling hologram. Two possible methods have been presented, i.e., SSM and SBMM, which provide similar outputs. We quantitatively calculate the accuracy of the background elimination, as shown in Fig. S1. The ground truth (GT) is the average of the calculated background from SSM and SBMM. With the increasing number of holograms in SSM, the NCC value turns out to be higher, and it reaches the highest point by using 43 of 75 holograms. By using SBMM, the NCC of the reconstructed background becomes higher with increase in the number of blocks, and it reaches the highest point when using 10 × 10 blocks by considering 75 total holograms in this calculation. The average NCC of SBMM is higher than that of SSM. However, SSM is faster than SBMM, as reported in Fig. S1. The computational time of SSM increases almost linearly with the number of superimposed holograms, and it reaches 10 s with 75 holograms. The computational time of SBMM remains almost constant at around 20 s with the size of blocks that divide the down-sampling holograms. Choosing the best parameters of SSM only takes less than one-third of the calculation time in SBMM.

**FIG. 1.**
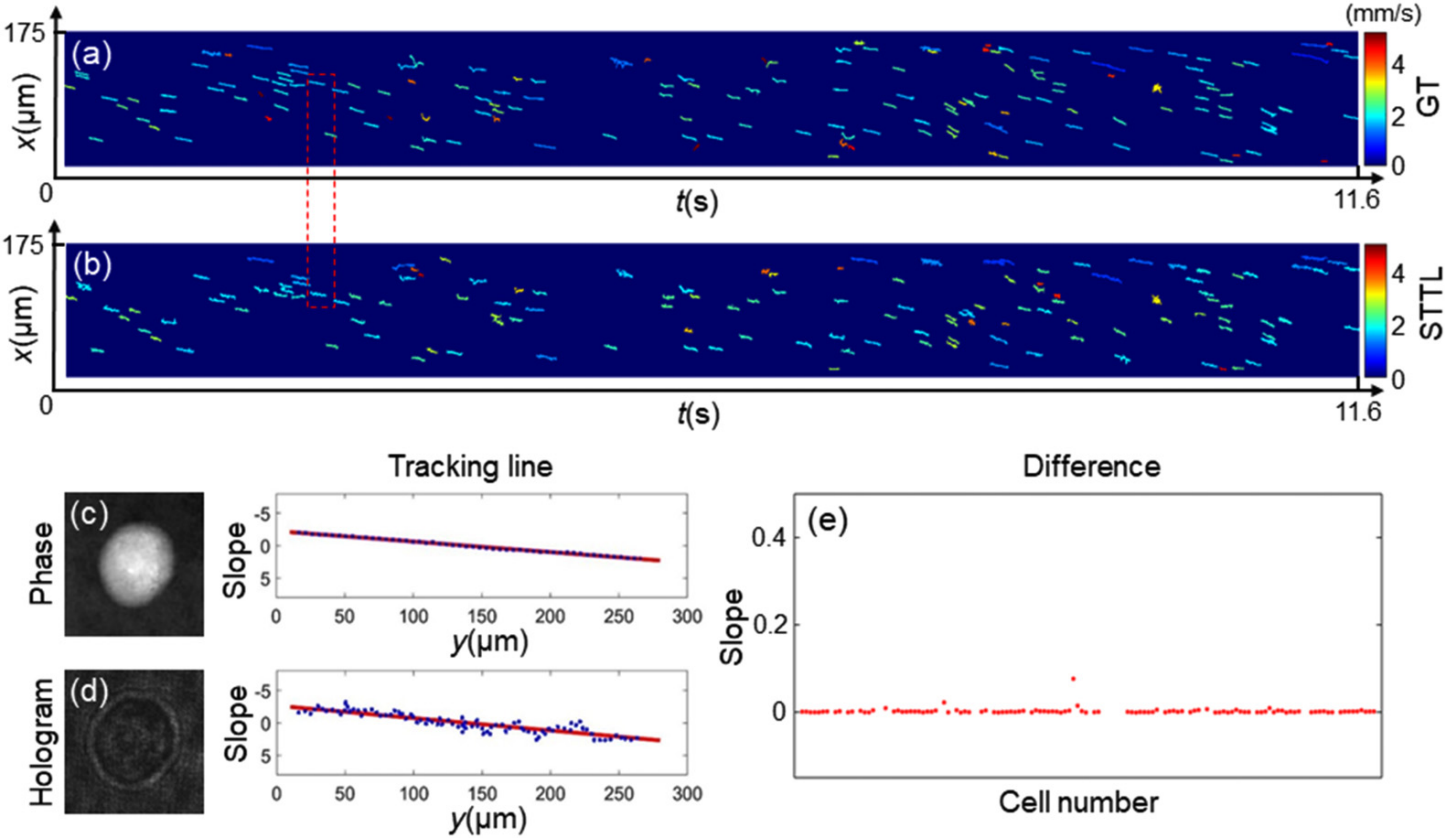
Performance of transverse localization. (a) and (b) STTL curves were computed over 15 s by using the conventional GT method and the proposed spatiotemporal tacking method, respectively. The total number of holograms is 11 618. GT: ground truth, STTL: spatiotemporal tracking line. (c) and (d) STTL curves of one flowing cell obtained from the QPM in the conventional GT method and from the hologram in the proposed spatiotemporal tacking method, highlighted in red in (a) and (b), respectively. The red solid line is a linear fitting of the actual positions (blue dots). (e) Difference between the slopes of the linear fittings about the entire analyzed cell computed by using the conventional GT method and the proposed spatiotemporal tacking method. The total number of cells is 131.

After selecting the SSM method for background elimination, Figs. 1(a) and 1(b) show the whole STTL over 15 s computed using the conventional method and the proposed strategy, respectively. The STTL from the conventional method can be regarded as GT. Figures 1(c) and 1(d) show the STTL of the flowing cell highlighted in Figs. 1(a) and 1(b), obtained using the conventional method and the proposed method, respectively. The visibility of the cell in the QPM in Fig. 1(c) is higher than in the case of the corresponding hologram without background in Fig. 1(d), thus the contour of the cell can be recognized more easily from the QPM. Nevertheless, all *x*-*y* trajectories computed by the proposed method exhibit high similarity with the GT. In fact, the blue dots in Figs. 1(c) and 1(d) represent the actual 2D positions, while the red solid line is the corresponding linear fitting. The comparison between the two transversal tracking outputs is displayed in Fig. 1(e) by the differences between the slopes of two linear fittings about all the flowing cells, which are almost null. The total number of cells is 131. However, it is worth underlining that there are some cases that are difficult to track from the down-sampling holograms, as summarized in the supplementary material. In fact, the difference between the STTL curves in Figs. 1(a) and 1(b) is displayed in Fig. S2(a), where some errors can be observed, which can be distinguished into two situations. The first one is the cells overlapping. In Fig. S2(b), two cells are very close to each other. However, while the cells are well separated inside the in-focus QPMs obtained after performing autofocusing through the conventional method, their diffraction patterns overlap in the out-of-focus holograms. Therefore, in the second case, the two cells are recognized as one cell. Hence, the red boxes in Fig. S2(a) show the two lines that come from the conventional method. Instead, the second error case is due to cells with weak phase values, as shown in Fig. S2(c). In this case, the weak cells are only visible in QPMs, while they are almost invisible in the holograms.

**FIG. 2.**
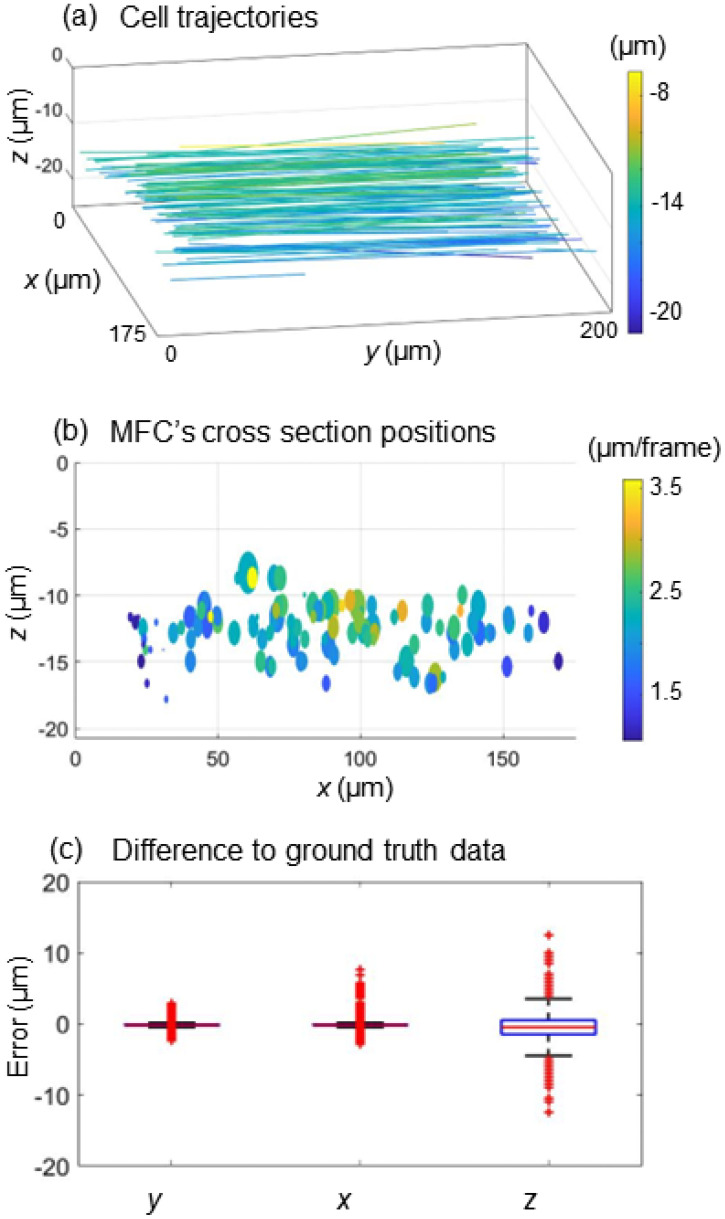
3D cell localization. (a) 3D trajectory diagram of all cells flowing along the MFC. (b) Cell positions are taken in the *x*-*z* cross section at the end of the MFC, with corresponding flow velocities in the colorbar. The circle's radius corresponds to the cell's equivalent radius. MFC: microfluidic channel. (c) Box plots of the error distributions about the *x-*, *y-*, and *z*-positions computed by the proposed method with respect to the GT. A total of 8432 positions are considered. Multimedia available online.
10.1063/5.0222932.1

After computing the *x*-*y* positions, the proposed STPS method is implemented to calculate the axial *z*-positions. Hence, the 3D trajectory diagram of all cells in the MFC is shown in Fig. 2(a) and Video 1 (Multimedia view), which corresponds to the 2D trajectories in Fig. 1(b). In particular, the 3D STTL curves are represented by their linear fitting. The *x*-*z* cross section taken at the end of the MFC is displayed in Fig. 2(b). Each circle is centered in the last position occupied by each cell in the *x*-*z* cross section, whose radius is equal to the cell equivalent radius, and whose color represents the cell velocity, according to the colorbar. All cells are concentrated in the^5,15^ micrometer range along the *z*-axis. To assess the performance of the proposed tracking method, 3D positions are computed also by the conventional method (i.e., the GT). Figure 2(c) shows the error distribution of *x-*, *y-*, and *z*-positions. A total of 8432 positions are considered in the error analysis. In particular, the localization errors are 0.214 ± 0.197 *μ*m for the *x*-positions, 0.264 ± 0.443 *μ*m for the *y*-positions, and 1.43 ± 1.208 *μ*m for the *z*-positions. Moreover, a comparison between the conventional method and the proposed method in terms of computational time is reported in Table I. For this analysis, 80 cells have been considered. For axial localization, the calculations are based on autofocusing of the reconstructed complex amplitude in the conventional method, and the calculation time from the hologram to the complex amplitude is included in 2D tracking. The computational time of the proposed STPS method is slightly higher than that of the conventional method. Instead, for the *x*-*y* positions, the computational time for the conventional method is ten times higher than that for the proposed method. The total computational time of the proposed method decreases up to 54% of the conventional one.

**TABLE I.** Computational times of the 3D cell tracking about the conventional and the proposed method evaluated over a dataset of 80 cells.

	2D tracking (*x, y*)	z tracking (s)	3D tracking (*x, y, z*) (s)
Conventional	174.49 s	150.14	324.63
Proposed	15.24 s	160.18	175.42

## III. CONCLUSIONS

In this paper, we proposed a spatiotemporal tacking method to speed up 3D holographic particle tracking. In fact, 3D holographic particle tracking is a fundamental issue among other necessary steps for cell diagnostics. The reported results mark the overcoming of the main constraints of conventional 3D holographic tracking. The spatiotemporal manipulation on holographic interference fringes is performed in place of the entire holographic reconstruction. In this case, the three-dimensional positioning of cells is divided into two independent processes: hologram-based horizontal positioning and STPS-based vertical positioning. While the 2D positions of each cell could be directly located by morphological calculation based on the reassembled hologram, the axial position is directly reconstructed by STPS complex-amplitude wavefronts. In this way, holographic reconstruction is avoided when the sole 3D cell tracking is requested. Experimentally, the proposed method reaches a negligible localization error lower than 1.5 *μ*m while only processing with 54% of the computational time. In particular, the main gain is obtained in the 2D transverse localization, which takes only one-tenth of the time required for the traditional method. In the expanded applications, introduction of the spatiotemporal tacking method can prevent holographic diagrams from being limited to traditional 2D image arrays. Taking advantage of the flow of the sample, the spatiotemporal digital hologram acquisition modality yields a substantially unlimited field-of-view and stores all volumetric data in a single continuous hologram. The 2D spatiotemporal tracking map compressed the 2D positions of cells and the time locations in the flowing period, achieving (2 + 1)-D tracking. By combining the STPS method, the *z*-axis positions of cells can be determined to achieve (3 + 1)-D tracking by only calculating in the spatial domain. Thanks to the ability for particle reconstruction in space and time domains simultaneously, this technique allows direct integration with a microscope and provides direct performance improvement of PTV by using low-cost components. A linear sensor array with a very high frame rate can replace a 2D camera to achieve high-throughput volumetric cell tracking and imaging. High-throughput volumetric reconstruction and single-cell analysis can be further expanded. The proposed approach can be used in microfluidics to analyze objects flowing in microfluidics channels. We believe that this platform could open new perspectives for enhancing the throughput by 3D rapid volumetric imaging.

## IV. METHODS

### A. HIFC recording system and conventional holographic tracking

**FIG. 3.**
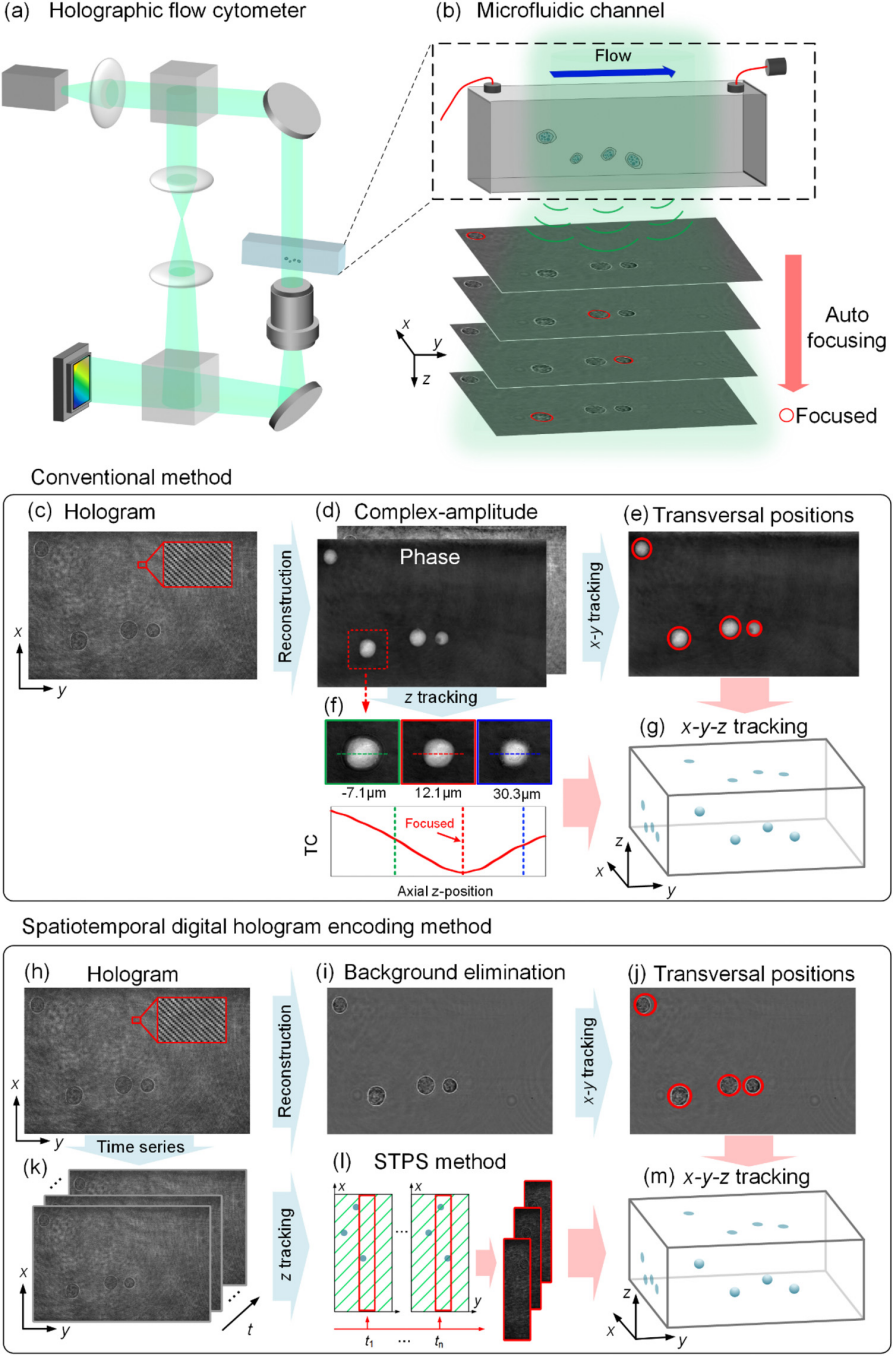
HIFC recording system and holographic tracking. (a) HIFC recording system is built based on off-axis digital holographic microscopy. L: lens, MO: microscopic objective, BS: beam splitter, M: mirror. (b) 2D axial projections of the 3D flowing cells in hologram. (c) The hologram of flowing cells. (d) The reconstructed complex amplitude from the hologram by filtering the object spectrum. (e) Transversal positions of each cell. (f) The TC values were calculated at different propagation distances for each single-cell region. (g) The x-y-z tracking results obtained by conventional tracking method. (h) The hologram of flowing cells. (i) The hologram after background elimination. (j) Transverse position of each cell. (k) A set of time-series holograms containing cell flow information. (l) Spatial-temporal phase shifting (STPS) method. (m) The x-y-z tracking results obtained by conventional tracking method.

The HIFC system sketched in Fig. 3(a) was built to rapidly record tumor cells flowing through the MFC.^46^ The experimental setup is based on an off-axis Mach–Zehnder interferometer.^47^ The cell type used in related experiments is HT1376. The medium is Minimum Essential Medium (MEM) with 10% fetal bovine serum (FBS). The biological sample is inserted in a syringe activated by a controlled microfluidic pump that realizes a flux at 7 nl/s through a commercial MFC, thus cells flow in different 3D positions. The DH configuration records the 2D axial projection of the 3D flowing cells, as shown in Fig. 3(b). The source is a coherent laser with a power of 100 mW and wavelength of 532 nm (MSL-U-532, China) and output through a single-mode fiber. The source is collimated by a lens (L) with a quasi-plane wave, and it is divided into an illumination arm and a reference arm by a beam splitter (BS). The wave illuminates the MFC to form the object wave. The object beam is captured by a microscopic objective (MO). The numerical aperture (NA) is 0.65 and the magnification is 40×. Two Ls are used to adjust the wavefront of the reference wave. The object beam and the reference beam converge through a BS, and the interference fringes are captured by the camera (5.86 *μ*m pixel size, 1200 × 1920 pixels), thus creating the off-axis digital hologram.

Holographic tracking methods allow reconstructing the 3D positions of each cell from a 2D hologram. Conventional holographic tracking methods are typically based on complex-amplitude reconstruction. The holograms need to be reconstructed frame by frame with a large calculation burden, thus increasing the total calculation time if long-term monitoring of flowing cells is required. The pipeline of conventional holographic cell tracking is summarized in Figs. 3(c)–3(g). The x-y plane location can be acquired by feature recognition, while the z-axis location is acquired from numerical refocusing.^12^ In off-axis holography, the object beam interferes with the reference wave with a slight angle between them. The interference fringe details of the recorded hologram are shown in Fig. 3(c). After Fourier transform, in the spectrum of the recorded digital hologram, the object wave, its conjugate wave, and the zero-order wave are separated; by filtering and centering the object spectrum, the complex amplitude of the out-of-focus cell can be obtained through an inverse Fourier transform.^29^ The QPM of the cell can be retrieved by extracting its argument and by performing phase unwrapping,^29^ as shown in Fig. 3(d). To select the region of the cell, edge extraction is applied in the unwrapping phase, and the cell contour is labeled in the binary matrix. From each cell contour, the centroid can be calculated, that is the 2D coordinates in the *x*-*y* plane, as shown in Fig. 3(f). Cell refocusing is performed by retrieving its axial z-position meaning that the complex amplitude is numerically propagated at different z-positions by the angular spectrum algorithm.^29^ For each of them, the image contrast is computed from the amplitude maps through the Tamura coefficient (TC).^43^ Minimization of the TC metric provides the in-focus z-position of the cell.^43^ In Fig. 3(f), three QPMs can be observed, and they are reconstructed at different axial positions, i.e., the in-focus position and two out-of-focus positions. Figure 3(g) shows the *x-y-z* tracking results.

### B. Spatiotemporal reassembly of holographic interference fringes allows to retrieve 3D cell locations

Conventional holographic tracking methods are typically based on the complex-amplitude reconstruction, as discussed in Sec. IV A. The holograms need to be reconstructed frame by frame with a large calculation burden, thus increasing the total calculation time if long-term monitoring of flowing cells is required. However, the spatial distribution of holographic interference fringes contains the wave intensity distribution and phase shift from the reference wave. Therefore, with the time continuity of the flowing cells, their phase shifting can be generated in the space domain.^49^ Reassembling spatiotemporal holograms is expected to improve the calculation efficiency of 3D tracking since holographic reconstruction is avoided.

**FIG. 4.**
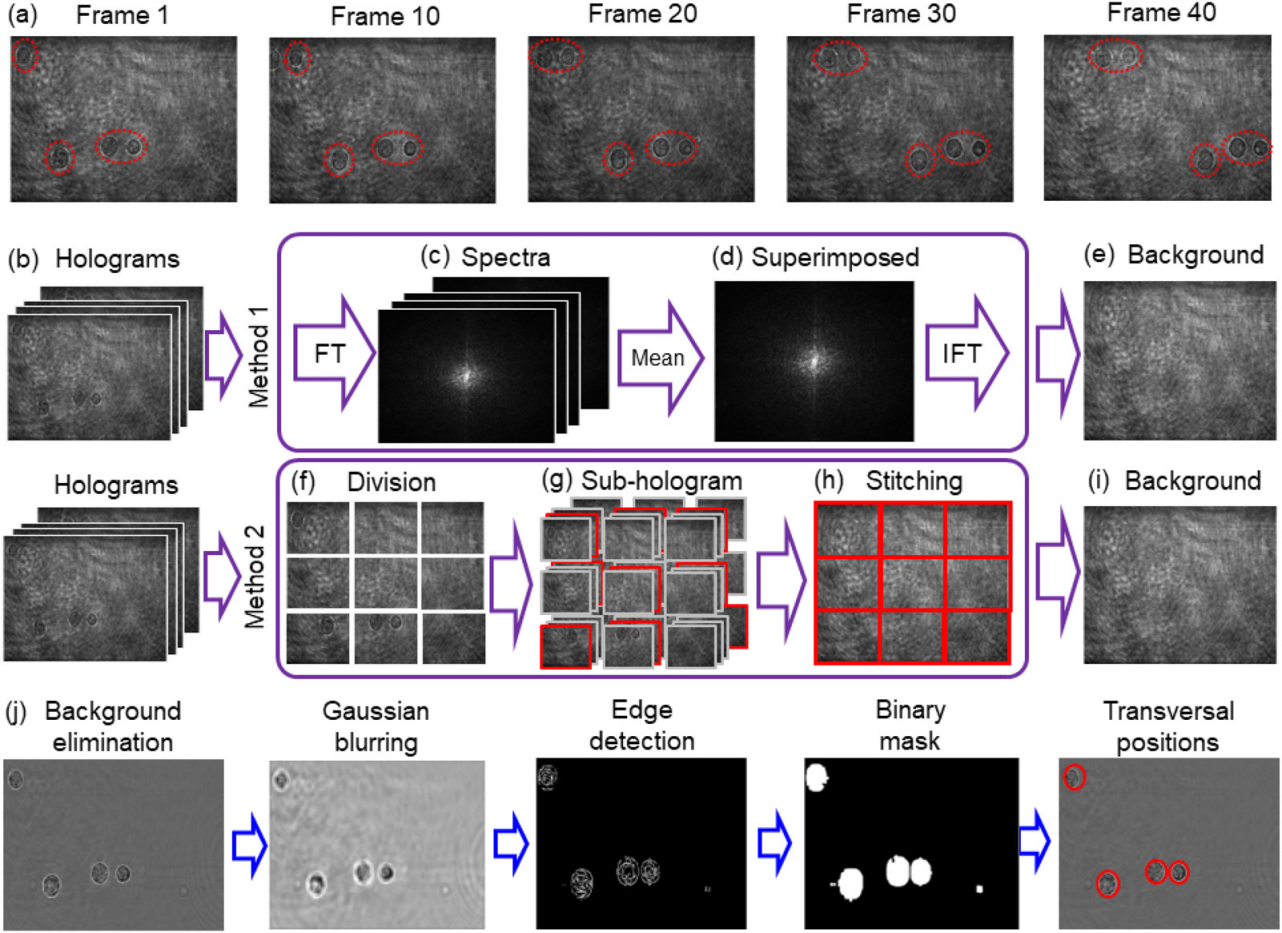
Transverse localization in a single frame. (a) Different frames of the down-sampling holographic video. (b) Down-sampling holographic stack. (c) Spectra of the down-sampling holograms and (d) corresponding superimposed spectrum. FT: Fourier transform, IFT: inverse Fourier transform. (e) Reconstructed background hologram obtained by performing the inverse Fourier transform from (d). (f) Division of one hologram in several blocks. (g) Division of the holographic stack in several blocks. (h) Background calculated from each block. (i) Reconstructed background hologram obtained by stitching blocks in (h). (j) Transverse localization based on the hologram after background elimination. The hologram has been processed by down-sampling.

First, we focus on the proposed transversal localization. As the cell is localized in the spatiotemporal hologram, the interference fringe pattern can disturb the contour of cells. The frequency of fringes is about half of the camera's sampling frequency. To eliminate obstruction from the fringes, holograms are down-sampled to half their original size. The number of pixels in one hologram is 1200 × 1920. After down-sampling, the number of pixels in one hologram is 600 × 960. Figure 4(a) shows the down-sampling holograms in different frames, in which the cell flow can be still clearly seen. However, the contrast of cells in the hologram becomes lower, which hinders the identification of cell contour. To obtain background-free down-sampling holograms and improve the contrast of cell contour, we propose two methods of background elimination. The first one is the superimposed spectrum method (SSM). In the HIFC system, the background is almost fixed in the holographic video while cells flow along the MFC. Therefore, the Fourier spectrum is computed for each frame of the holographic video, and a new spectrum is created by the average operator, as shown in Figs. 4(b)–4(d). In fact, when we superimpose all the spectra, the spatial frequency of the quasi-static background is strengthened while the spatial frequencies of flowing cells become average and weakened. By performing the inverse Fourier transform of the superimposed spectrum, the background hologram can be reconstructed, as shown in Fig. 4(e). Another background elimination method is the static background matching method (SBMM). All the holograms are divided into many blocks, as shown in Fig. 4(f). The flowing cells hold sparsity in the whole FOV. The consistency between adjacent frames becomes high when there are no cells flowing through this block. We can calculate the Normalized Correlation Coefficient (NCC) between adjacent frames of all blocks to find the adjacent frames with high consistency,^48^ which can be realized as the background. When there are cells flowing through the block, the NCC becomes lower because the positions of flowing cells change in a different frame, as shown in Fig. 4(g). In the calculation, we set the threshold value of NCC as 0.98 to distinguish the background. The performances of the background elimination methods are shown in the supplementary material. After calculating all blocks, they are stitched with each other, as shown in Fig. 4(h), thus obtaining the background in Fig. 4(i). The two proposed methods correspond to situations with different cell concentrations within the channel. When the cell concentration is low, SBMM is recommended; when the cell concentration is high, SSM works better. The related descriptions have been presented in the revised manuscript. After calculating the background, all down-sampling holograms are divided by it to achieve background-free down-sampling holograms [see Fig. 4(j)]. Then, the *x*-*y* tracking is based on this new holographic stack. Normalized holograms are low-pass filtered by Gaussian blurring. Edge detections are applied to create a binary mask for each cell. Finally, the cell centroids are calculated as 2D positions in the *x*-*y* plane.

**FIG. 5.**
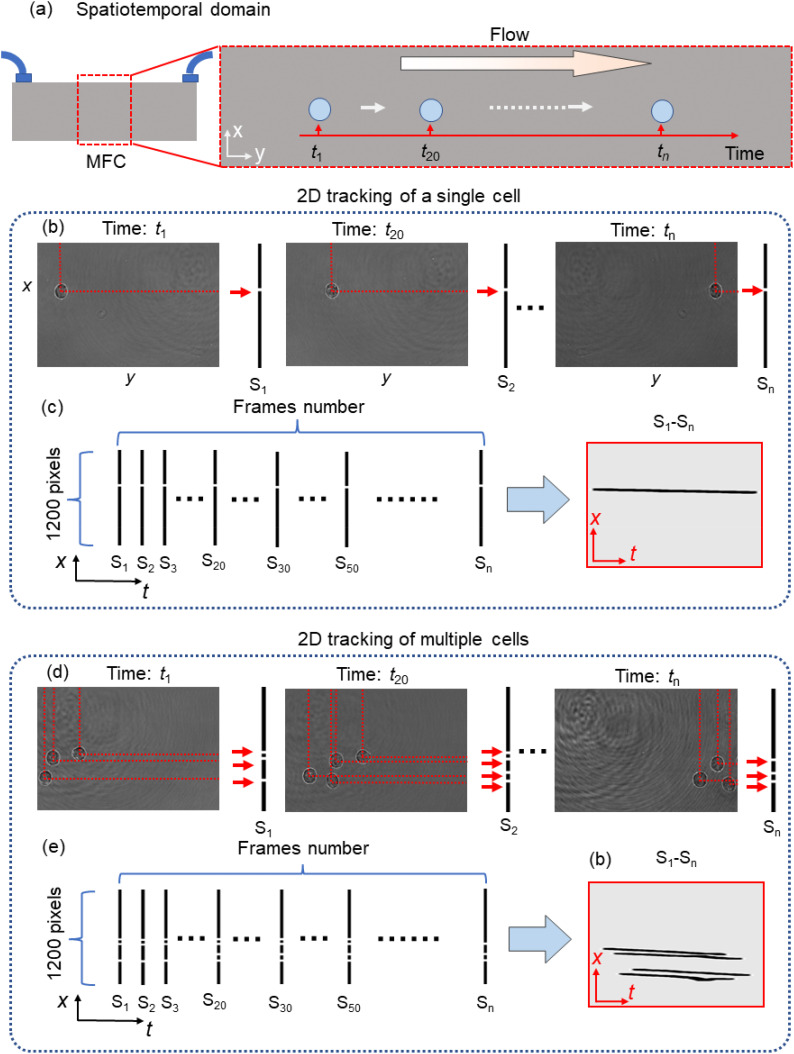
Spatiotemporal transverse localization. (a) The spatiotemporal domain of flowing cells. MFC: microfluidic channel. (b) Calculation of the *x*-positions of a single cell in each frame. (c) STTL of a single cell obtained from the 2D spatiotemporal trajectory. (d) Calculation of the *x*-positions of multiple cells in each frame. (e) STTL curves of multiple cells obtained from the 2D spatiotemporal trajectories.

Figure 5 shows the spatiotemporal location method of the flowing cell. The spatiotemporal tracking line (STTL) is constructed to show the flowing cells. In Fig. 5(a), one cell is sketched in its continuous flow inside the MFC, with the three highlighted positions corresponding to different times. For each frame, the 2D *x*-*y* positions are calculated by the method shown in Fig. 4. To process the holographic video sequence, we construct a spatiotemporal calculating framework of flowing cells. For single-cell tracking, the *x*-position is labeled in one-column pixels, as shown on the right side of each frame in Fig. 5(b). All holograms containing this single cell are processed, and the *x*-column pixels of all frames in Fig. 5(b) are combined to create the STTL of that cell, as shown in Fig. 5(c). The sequence number of the hologram is directly related to the flowing time, which makes up the time coordinates. The *x*-*t* STTL reveals the spatiotemporal flow of the cell along the MFC. The variables from S_1_ to S_n_ represent the labeled one-column pixels from all holograms. For tracking multiple flowing cells in the FOV, multiple positions in the *x*-axis are labeled in one-column pixels, as shown in Fig. 5(d). Then, multiple *x*-*t* STTL curves are reconstructed, as displayed in Fig. 5(e). The spatiotemporal tracking map indicates the situation of flowing cells during the whole monitoring period. It intuitively reveals the evolution of cell density distribution and the 2D relative positions of the cells inside the MFC. Considering the approximate uniform motion of cells in the MFC, the y-axis position of cells can be analyzed with y = vt, where v is the average flowing velocity. The 2D spatiotemporal tracking map compresses the 2D positions of cells and the time locations in the flowing period, achieving (2 + 1)-D tracking.

**FIG. 6.**
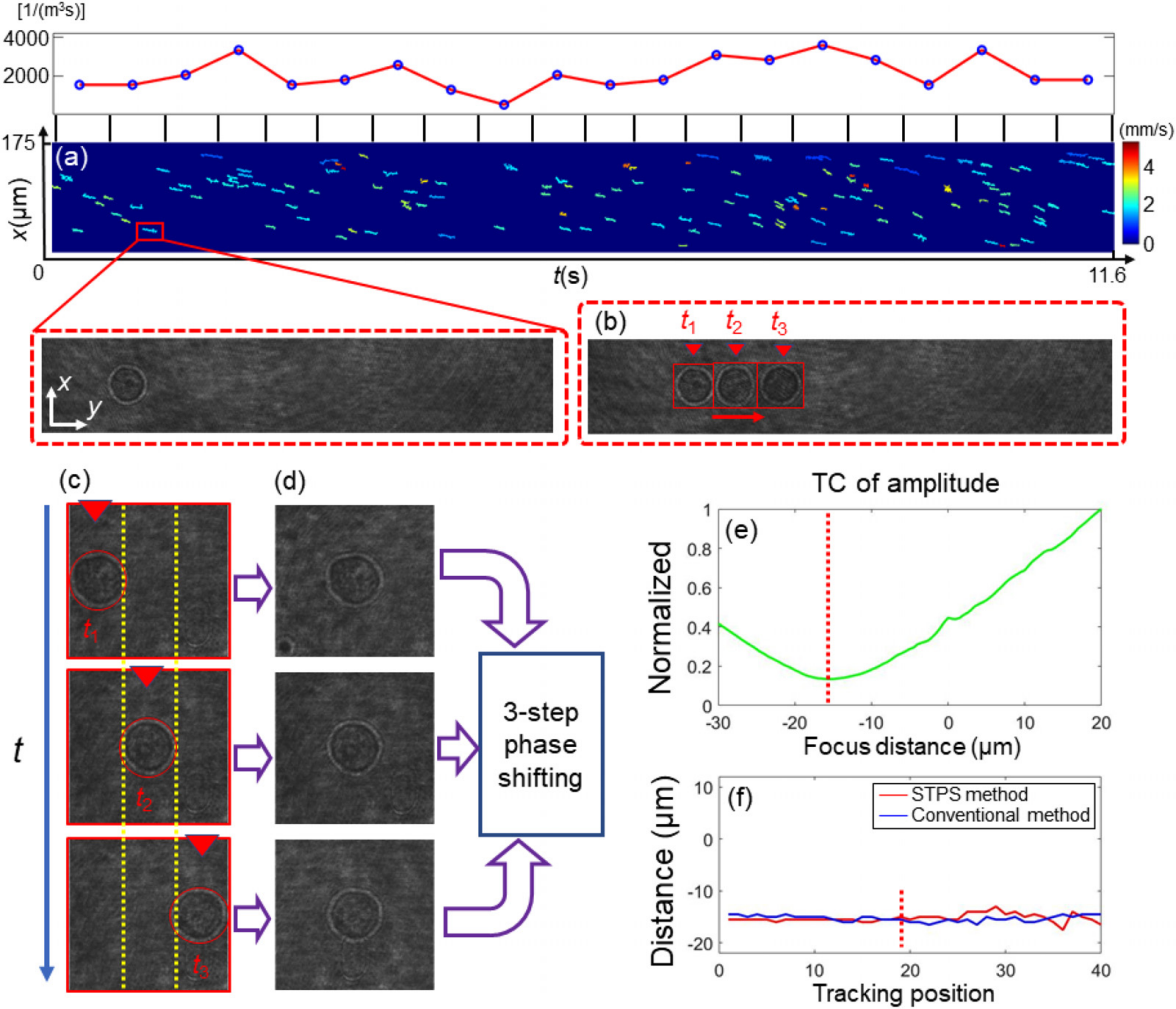
The calculation of *z*-positions by STPS method. (a) STTL curves of all the cells flowing along the MFC, with one cell highlighted in red inset. The throughput of flowing cells at different time intervals is shown above. (b) and (c) Three frames when the cell flows the distance of the size of the cell. (d) Centered ROIs computed from the holograms in (c). (e) TC values at different z-positions by performing numerical propagation based on the complex amplitude from the STPS method. TC: Tamura coefficient. (f) In-focus axial positions are computed by the conventional method (blue) and the proposed method (red).

By using spatiotemporal tracking, the 2D STTL under the whole monitoring period can be constructed, as shown in Fig. 6(a). The number of pixels in one hologram is 1200 × 1920. The total number of holograms is 11 618. The size of the STTL matrix is 1200 × 11 618. The lines in Fig. 6(a) encode the *x*-positions and the corresponding times the flowing cells appeared in the imaged FOV. Hence, the STTL matrix compresses the flowing speed and the concentration of flowing cells of the whole holographic video into one spatiotemporal matrix. The flowing time of each cell in the imaging FOV can be calculated according to the length of each line. Hence, we can further calculate the average speed of each cell based on the length of the imaged FOV (i.e., the space) and the length of the STTL curve (i.e., the time). The average flowing times in the imaged FOV are encoded with different colors in Fig. 6(a). Moreover, from the 2D STTL, it is convenient to calculate the concentration of flowing cells in the volume of the MFC corresponding to the imaged FOV. The boundary of MFC is almost the same as the FOV. The number of pixels in imaging FOV is $N_{x}\times N_{y}$ with 1200 × 1920 pixels, and the magnification is 40, the pixel size Δ is (5.86/40) *μ*m. The whole volume of the MFC in the imaged FOV can be calculated as $\pi {\left(N_{x}\Delta /2\right)}^{2}N_{y}\Delta$ by considering the cylindrical shape of the MFC and the area of the FOV. The sampling time is assumed as $\Delta_{t}$ = 0.998 ms, and the number of frames is $N_{t}$ = 11 618, then the monitoring period is $N_{t}\Delta_{t}$ = 11.6 s. We assume that the number of observed cells is $N_{c}$, then the cell throughput can be calculated as $N_{c}/\left[\pi {\left(N_{x}\Delta /2\right)}^{2}N_{y}\Delta N_{t}\Delta_{t}\right]$, which is defined as the number of cells passing through the MFC per unit volume and per unit time, and the unit is $1/\left(m^{3}s\right)$. We divided the whole monitoring period into 20 equilibrium intervals. As shown above in Fig. 6(a), the cell concentration changes in each interval.

After discussing the transverse localization, the proposed axial localization method is introduced. The z-positions are located by the STPS method. To perform STPS, the STTL curve of a single cell is first computed. In fact, the three frames in which the cell has moved a length equal to its diameter are selected, as displayed in Figs. 6(b) and 6(c). Figure 6(b) represents the corresponding three moments in the cell's flow. The three cells are obtained from three moments, and they represent the flowing process of cells. In these three frames, the cell contours are tangent to each other. A region of interest (ROI) is centered around the cell in these three transverse positions, as shown in Fig. 6(d). The three holographic ROIs can be expressed as $U_{1}\left(x,y,t_{1}\right)$, $U_{2}\left(x,y,t_{2}\right)$, and $U_{3}\left(x,y,t_{3}\right)$. Essentially, this is a three-step phase-shift process for spatiotemporal interference fringes; in our previous study, it has been discussed in detail.^49^ We have

$$E=\frac{1+i}{4}\left[\left(U_{1}-U_{2}\right)+i\left(U_{3}-U_{2}\right)\right].$$ (1)

Once the complex-amplitude field E is retrieved, the TC discussed in Sec. IV A can be applied to retrieve the in-focus z-position. Here, spatiotemporal phase-shift allows for assembling an incomplete cell complex-amplitude information, which is sufficient for the z-position retrieval of the cell but cannot be used to reconstruct a valid internal information. Figure 6(e) shows the TC values of the amplitude at different axial positions. The cell axial position can be determined by the minimum value. Three holograms are used to calculate the z-axis values. The corresponding z-axis position is the position of the middle hologram. Figure 6(f) shows the comparison between the z-positions of the analyzed cell computed by the conventional (blue) and the proposed (red) methods. Almost all positions are consistent with traditional methods.

## SUPPLEMENTARY MATERIAL

See the supplementary material for a detailed description of background elimination and transversal localization.

## Data Availability

The data that support the findings of this study are available from the corresponding authors upon reasonable request.
